# Mr. Jardine's Case of Collapsed Brain

**Published:** 1805-10-01

**Authors:** Wm. Jardine

**Affiliations:** Dumfries


VOL. XIV.] OCTOBER 1, 1805. [NO. LXXX.

*Printed for R. PHILLIPS, by W. Thorne, Red Lion Court, Fleet Street, London.*

### *To the Editors of the Medical and Phyſical Journal.*

GENTLEMEN,

THE enclosed case of a collapsing state of the brain, of a man whom I trepanned on board his Majesty's ship Victorious, being, as I think, as interesting to the physiologist as the surgeon, I request the favour of you to allow it a place in your Journal; and if a case thought worthy of such attention, I may probably send you some others. I am, &c.

*Dumfries, August* 25, 1805. WM. JARDINE.

---

On the 1st of August, I was sent for to John Gray' aged 46, who the night before fell down the main hatchway. As he did not immediately after the accident complain, or appear any way hurt to those who took him up, being drunk, he was as soon as possible put to bed, where he remained until the morning; when his mess-mates seeing he could not be awoke, at first thinking him only asleep, began to be apprehensive of something more being the matter. I found him in a very stupid lethargic state, attended with some of the other symptoms of a compressed state of the brain.

In examining his left ear, to which some drops of dried blood in its cavity had directed me, he cried out very passionately, expressive of much pain, though before he could not be made to speak. There was the mark of a blow on the occiput, yet I could not discover, from all I saw, enough to satisfy me with respect to the very place where an operation might most successfully be performed for his relief. He was plentifully bled, and his head after being shaved, bathed with a cooling solution of the cerussa acetata; he was likewise ordered an injection, which, however, soon after insensibly came away, as also did his urine. The ear seemingly injured was cautiously syringed with warm water, with the view of promoting a discharge that way; a practice I have, in a few similar cases, formerly had recourse to with some advantage; but that operation rather paining him, without answering its end, was left off.

Nothing could be given him by the mouth

5th. All the right side paralytic,* and in every other respect worse. On examining his head again, it was at last observed, that in feeling a particular part, betwixt the left ear and occiput, he moved his head a little, and attempted to raise his hand, and seemed to feel what he did not in examining the same place before. This place therefore I considered to be the most proper for applying the saw; and in dividing the teguments, I was the more satisfied in what I had done, from nearly the size of a shilling of the pericranium, directly under, being dusky and varicose, and not so adherent as it should be.

As soon as the piece of bone was taken out, a thick congealed blood, seemingly within a very fine membrane, and partaking of the pulsation of the brain, rose at the perforation, and was, as I afterwards found, collected in such a quantity within the skull, as to separate the dura mater from it the whole extent of the parietal bone, and was so elastic and adherent to that membrane, it was with much difficulty, after penetrating almost an inch deep from the edge of the foremost part of the perforation, I could extract about two ounces. I thought it, therefore, most adviseable, observing his senses returning, to desist and wait the effect of time, and the operation upon that matter, which even at that time, by another opening, coul not have been completely cleared out.

The dressings were very simple, and he was kept very quiet.

In dividing the teguments he was very restless, but did not show the least sensibility or feeling, in introducing the instrument under the cranium. About an hour after the operation he seemed much relieved, knew his messmates, and asked for a drink, and his pulse became more regular.

6th. Had a good night's rest, and surprisingly better, his appetite &c. returning. Though he sometimes spoke incoherently

* Whether he was in that paralytic state the day before, I do not now recollect, that circumstance not being noticed in my Journal. I rather think, from the way he lay he was, but it must have escaped my observation.

coherently and to himself, when spoken to he gave a rational answer. His pulse very regular in the forenoon, but a little quickened in the afternoon. No discharge of the compressing matter that we could expect any sensible benefit from.

7th, 8th, and 9th. In every respect gradually getting better. His pulse always quick in the afternoon.

The compressing matter nowise altered in its consistence, very little at a time therefore, these three days past, could be taken out; but by the little that was, he was always sensibly lightened. The morning of the 9th rather low, in consequence of a profuse hæmorrhage from the membranes within the cranium, and likewise from the teguments; he was therefore allowed a little wine in his sago; a poultice was applied over the dressings on the eighth, but on account of the hæmorrhage of the 9th, it was laid aside.

10th. Continues better. The dressings were not removed on account of the hæmorrhage the day before. Requested a little more wine in his sago, of which he took very plentifully.

11th. The compressing matter still very tough and difficult of extraction, but a great discharge of a brown sanies from the membranes of the brain; the teguments all firm and sound. Had the bark with wine.

12th, and 13th. The compressing matter not being so cohesive as before, a considerable quantity of it was brought away; and there was such an increase of the same ichorous kind of discharge as yesterday from the membranes, that it run through all the dressings. The dura mater, of a dark brown colour, for the first time, made its appearance; was very firm, and was again with the brain apparently rising to fill up the space they formerly occupied. No pulsation of the brain observable on the 12th, except at a particular point near the edge of the perforation; his pulse still quick in the afternoon; however, he was in all other respects gradually getting better.

Ordered as much bark as his stomach could well bear, wine, &c.

14, 15, 16, 17. The compressing matter now coming away very freely, accompanied with a vast discharge of the sanies from the membranes. The dura mater in appearance more healthy; on the 14th some red spots or granulations were observed upon it; and by the 17th, all opposite the perforation was of a fleshy appearance. The pulsation of the brain being very weak, was scarcely ob-

 servable, servable, unless the membrane was touched by the probe; but it was gradually getting stronger. Every thing externally looked well. Continued the bark, wine, &c.

Between the 18th and 21st. A profuse hæmorrhage from the membrane the 18th, that depressed him that day very sensibly, but on the 19th quite revived. The discharge of the compressing matter daily diminishing, but that from the membrane increasing, and becoming more offensive. In the afternoon his skin very hot, and his pulse as before, always quick, but in all other respects as well as could be expected, and had always a very good appetite. Medicines as before.

22d and 23d. Both nights a profuse bleeding from the inside of the cranium, and also a considerable increase of the sanious discharge from the membrane, but he did not sink under such a loss, so much as might have been expected, and he very soon got the better of it. A further increase of wine, with the bark, &c.

24th. The sanious discharge of the membrane as profuse as ever. The brain and membrane now risen, almost all round, equal with the lower part of the perforation: The membrane not so lively in its appearance as the day before. To day I found he seldom received the bark as directed, and indeed had taken very little, to which neglect may be attributed the continuance of such an excessive and offensive discharge from the membrane. Continued the bark and wine.

Between the 25th and 31st. The brain and membrane now risen close up to the edge of the perforation, and on the 27th the membrane was beginning to attach itself to the bone. The ichorous discharge from it diminishing daily; not so offensive in the smell, and thicker. The pulse in the afternoon not so quick as before. The pulsation of the brain on the 30th scarcely observable. On the 31st it was stronger, and the membrane where it was forming an adhesion to the bone was withdrawing itself from it, the air forcing its way through the opening made by that separation. It appeared as if the brain had been sinking in and drawing the membrane, which indeed was but slightly attached to the skull at that time, except at the back part, along with it.

Betwixt the 1st and 5th of September the brain and membrane continued gradually sinking in; by the 5th it had shrunk at least a quarter of an inch from the lower edge of the perforation, whereby such an opening was made made that I could introduce the probe, in a direction forward, about two inches and a half betwixt the membrane and cranium. From such appearances I was satisfied that an attachment of the membrane to the inside of the cranium, but in a very slight or partial manner, ever had taken place. The instrument introduced, altho' covered with a cotton thread, did not show matter of any kind betwixt the cranium and membrane. The teguments were firm and adherent, and he had all the appearances of returning health and strength. He took about ℥iiß of the bark every day, and was liberally supplied with wine, and a large blister was applied to the back of his neck.

From Sept. 6 to Sept. 9. The brain and membrane again gradually rising up to the edge of the perforation. In introducing the probe into the cranium, it was every day more and more confined in its room to move in, so that by the 9th, it had not the eighth part of an inch. The membrane looked well, and was again supposed to be forming an attachment with the cranium. No kind of discharge except a few drops of a thin colourless fluid that run out on the 7th and 9th, but the part of the dressings (a piece of lint) opposite the perforation was blackened every day by a kind of imperceptible vapour from the inside of the cranium. The brain rather in a dormant state, sometimes a slight pulsation was excited by touching the membrane, at other times when touched it seemed quite insensible.

As I imagined the separation of the brain and membrane from the cranium might have been somewhat promoted by the erect posture of his body, having sat up very much in the course of the day, (the 5th) he was on the 6th and 7th, the two following days, ordered to lie down; but the change of posture I found to have no observable effect in promoting the adhesion of the membrane to the cranium, no particular situation therefore afterwards was attended to.

Had a quart of Madeira wine, besides ℥iiß of the bark every day, and was also plentifully supplied, having always a good appetite, with fresh meat, soups, &c.

From the 10th to the 14th. The brain again gradually receding from the perforation, and the most of the time rather in a dormant state; a pulsation only sometimes could be excited even when the membrane was touched. The probe, which on the 9th could not be introduced the eighth of an inch under the cranium, now easily found its way as far as at any time before. By introducing it on

 the the 11th, about two tea spoonfuls of the same colourless fluid was discharged as on the 7th and 9th. On the 13th, a pulsation of the brain was occasioned, which forced out a little more of the same kind of fluid as on the 11th. On the 14th the discharge thicker, and the lint again blackened by the vapour from within.

15th. The brain now shrunk more than a quarter of an inch from the perforation, and in such a torpid state as not to be roused, even when the probe was introduced under the cranium as far as the sagittal or coronal suture. In touching the inside of the cranium with the probe, I thought I felt some parts of it softer than others. Nothing whatever discharged. His hand in the course of the day sometimes shook very much; he had taken unusual exercise the day before. The bark was given him with mustard-seed, and his allowance of wine.

Betwixt the 16th and 20th. The brain and membrane again gradually rising, but still in a very inactive state. A weak pulsation only observable on the 20th, except three slight strokes, which were occasioned by touching the membrane on the 17th. No kind of discharge before the 20th, when by laying him upon his back, a little purulent matter was forced out by the pressure of the brain against the cranium. The integuments all sound and firm.

The mustard-seed in every form disagreeing with him, was left off. Continued the bark and wine as before.

From the 21st to the 23d. The dura mater looked well, and seemed to be getting firmer in its attachment to the bone, and more likely than ever with the brain to maintain its proper situation. The pulsation too more lively than for days past; he was put into the same recumbent situation as on the 20th, with the design of forcing up any matter that might again be lodged betwixt the membrane and cranium, but nothing appeared. The same treatment continued.

From the 24th to the 26th. The brain and membrane again sinking in, and the opening betwixt the membrane and cranium again so wide as to admit the probe easily half an inch. The pulsation of the brain only observable on the 24th, and then very weak. The lint applied to the perforation the 25th and 26th, was wetted with arrack, and a stimulant sinapism was applied to the right, or formerly paralytic foot. Bark and wine.

From the 27th to the 29th. The brain again rising. On the 29th, rather prominent, and the membrane seemingly completing its adhesion with the bone.

The

The spirit applied on the 25th and 26th felt cold. On the 27th the spirit. terebinth. was therefore tried; but the second application of the terebinthina, on account of its occasioning a great heat in the left side of the head, was taken off. The sinapisms were continued, but irritating him very much and preventing his sleep, at night they were taken off. Bark and wine.

30th. An opening again made by the separation of the membrane from the skull, indicated the collapsing state of the brain.

Between Oct. 1 and Oct. 4. The brain and its membrane again gradually rising.

Continued the bark, wine, sinapisms, &c. and renewed the blister at the back of his neck.

Between the 5th and 9th. Although the membrane was firmly attached to the bone, a concavity at the perforation showed that the brain was again in a collapsing state. From the 7th the spirit. terebinth. was applied externally to the place perforated. Besides the bark and wine, he drank very freely of valerian tea, or had the bark mixed with it.

Betwixt Oct. 11 and Nov. 11. The place perforated, although concave, was very firm, except at the foremost part, where a small crust was formed on the place where that matter had burst out before. The ossification was therefore thought very slow or doubtful; however, on November 11, all the dressings were laid aside, and a firm substance was fixed in his cap, to defend the place from external injury, and he returned to his duty, which he did without any inconvenience whatever, until the night of September 5, when he was taken with a head-ach, which he was relieved of the same night, by a quantity of purulent matter bursting through at the foremost part of the place perforated, where the adhesion of the membrane had all along been so slight. The quantity discharged, from what I saw and was told, I judged to have been about an ounce and a half.

Betwixt Dec. 6 and Dec. 21. The discharge lessened gradually to about a tea spoonful every second day, when it was dressed, and became quite clear.

Betwixt Dec. 21 and Jan. 13. No kind of discharge whatever or the least appearance of it upon the dressings. No pulsation or feeling from introducing the instrument under the cranium, until three days before the place closed up, when he felt a little more than he had at any

 time time before. The convexity at the perforation continued, but was very firm except at the foremost point, which was only covered as before, by a kind of crust that was forced off on Feb. 11, by some purulent matter bursting through a hole about the size of a crow-quill, which discharge relieved him of a slight head-ach he had the night before. In introducing the probe on February 12, a small quantity of mixed matter was discharged, and the membrane, except at the edge of the perforation, was found to be as extensively detached from the cranium as ever. No pulsation at that time observable. It continued open until the 18th, without so much as an appearance of having discharged any thing whatever, except what was observed upon the lint, that was blackened as in the preceding stages of the injury.

A whitish thin matter burst out again on February 24, but the discharge from that time to March 6 being very trifling, the place was looked at very seldom.

Betwixt March 6th and 16th. No discharge of any kind whatever. When the dressings were taken off, a pulsation was sometimes observed at the orifice, which ceased as soon as the probe was introduced under the cranium. The lint upon the orifice sometimes black and sometimes brown.

October 17, 1800. Seven months after the orifice on the edge of the perforation was closed up by a firm incrustation, it was again opened by a pale thin matter, which, from the appearance of the dressings, could not have exceeded half an ounce.

The matter did not alter its consistence before the 24th, when it became whiter and thicker, and diminished until the 27th, when the orifice closed up, and by November 1, became very firm.

Before the 27th I could not introduce the probe a quarter of an inch, and he was then more sensible of the touch, and complained more than ever he did before.

Betwixt Nov. 1, 1800, and Feb. 13, 1801. All very well. On Feb. 4 again complained of a head-ach, which was, as sometimes before, relieved by a pale thin matter bursting through at the foremost part of the place perforated.

The sensibility now so increased that it was with much difficulty I could introduce the probe a quarter of an inch, or even touch the orifice without giving him much pain. The discharge diminishing gradually, it healed up again Feb. 16.

On the 5th of August following, the last time I saw him, the the place perforated was very sound, although externally concave. He never from that time experienced any other uneasiness from the injury in doing his duty, unless he had caught a cold, when coughing or sneezing made his head ache.

It may be necessary to mention that this man was subject to an obstinate costiveness, for which he was obliged to take laxatives almost every second or third day, and that he suffered very much from his stools and urine insensibly coming from him, and that it was not before Sept. 5, a month after the accident, that he got the better of that disagreeable complaint.

What particularly struck me in this man's case was, that the brain, whether in a collapsing, inactive, or more irritable state, did not observably influence the system in general, or was at any time accompanied by any of thsoe symptoms denominated nervous; and that the brain and membrane seemed so firmly attached to one another, that they rose and sunk together, unless where the attachment of the membrane was very firm to the cranium, backward, the brain in collapsing was sure to tear it from it.

That he never was sensible of the membrane being touched, or of the probe or any other instrument being introduced betwixt the membrane and the cranium, although at that time a pulsation of the brain, when in a dormant state, was sometimes excited by it, until near about his recovery, when the sensibility of the parts touched became very quick.

That the loss of blood he met with at four different times, which at first, I dare say, exceeded twenty ounces, and at different times afterwards as much more, observably had no effect in retarding the recovery of his paralytic side. Mr. Hill (if I recollect) mentioned a case of a man, who after recovery of a paralytic state of his head, occasioned by a blow, relapsed in consequence of a subsequent discharge of blood from that part.

That though the dura mater was so long detached from the skull the teguments were always very firm and adherent, and never had any thing like an unhealthy appearance, except for a few days between the 20th and 30th of September, when a space, rather larger than a sixpence at the lower corner of the incision, appeared rather spongy, and from which place about a tea spoon full of purulent matter, lodged between the skin and cellular substance, was pressed out.

The membrane being sooner and more firmly attached to to the back and lower pait of the perforation, than the fore and upper, was obviously owing to the perforation being nearer the lambdoidal suture, and by the brain, probably by its weight, acting against the side of the cranium, thereby forcing up any matter likely to collect in the lower part of the cavity, by which the membrane was kept in closer contact with the bone.

The bark, when properly given evidently, was very effectual in correcting the irritable quality of the discharge from the membrane, and drying it up; but neither it nor any thing else he took afterwards, with the same intention, had any observable effect upon the brain, either in its dormant or more irritable state, or appeared to me to be of any benefit to him whatever.

---

